# Unraveling COVID-19-vaccination-induced bullous pemphigoid: a case report and review of the literature

**DOI:** 10.1186/s13256-025-05652-x

**Published:** 2025-11-28

**Authors:** Savitri Chandrasekaran, Mamta Kamboj, Snigdha Baddepudi, Rishu Raj, Chaitanya Suresh, Vivek Sanker, Tirth Dave, Nishan Wijenaike

**Affiliations:** 1 West Suffolk Hospital, Bury St Edmunds, UK; 2 Government Medical College, Patiala, India; 3 Dayanand Medical College and Hospital, Ludhiana, India; 4 University Hospital Southampton NHS Foundation Trust, Southampton, UK; 5 Atal Bihari Vajpayee Institute of Medical Sciences and Dr. RML Hospital, New Delhi, India; 6 James School of Medicine, The Quarter, Anguilla; 7 Department of Neurosurgery, Stanford University, Stanford, CA USA; 8 Bukovinian State Medical University, Chernivtsi, Ukraine

**Keywords:** Bullous pemphigoid, Vaccines, Dermatology, COVID-19, Rash, Case report

## Abstract

**Background:**

Coronavirus disease 2019 vaccines have been instrumental in combating the global pandemic, yet their potential side effects, including autoimmune conditions such as bullous pemphigoid, remain an area of concern. This case highlights the development of bullous pemphigoid following coronavirus disease 2019 vaccination and includes a comprehensive review of similar cases reported in the literature, emphasizing its novelty and clinical significance.

**Case presentation:**

An elderly British man in his 80s with type 2 diabetes mellitus developed blistering lesions 21 days after receiving his third dose of coronavirus disease 2019 vaccine (Moderna). Clinical examination revealed erythematous plaques and bullae on the trunk and limbs. Histopathological evaluation and immunofluorescence confirmed the diagnosis of bullous pemphigoid. Treatment included corticosteroids, doxycycline, and immunosuppressants. Despite initial improvement, a severe flare-up necessitated hospitalization and wound care management. A systematic review identified 50 reported cases of bullous pemphigoid linked to coronavirus disease 2019 vaccination, with the Pfizer-BioNTech vaccine implicated in most cases (64%), followed by Moderna (18%). Symptom onset typically occurred after the first dose in 52% of cases.

**Conclusion:**

This case underscores the need for vigilance regarding autoimmune phenomena such as bullous pemphigoid following coronavirus disease 2019 vaccination. Awareness of such potential adverse effects is crucial to ensure timely diagnosis and management, ultimately contributing to patient safety and guiding future vaccine development.

## Introduction

The global pandemic of coronavirus disease 2019 (COVID-19) has posed an unprecedented challenge for healthcare systems worldwide, leading to significant morbidity, mortality, and socioeconomic disruption [1]. In response, multiple COVID-19 vaccines were rapidly developed and deployed to mitigate disease spread and severity. As these vaccines are administered on a large scale, close monitoring of adverse events is crucial, particularly those that may result in long-term complications.

While COVID-19 vaccines have been highly effective in preventing infection and reducing associated complications [2], emerging reports suggest a potential link with autoimmune conditions in some individuals, as well as exacerbation of existing autoimmune diseases [3–6]. Bullous pemphigoid (BP), a subepidermal autoimmune blistering disorder, has been observed as a rare reaction following vaccination. In addition to autoimmune phenomena, various cutaneous adverse effects have been documented, including lichen planus, psoriasis, cutaneous small-vessel vasculitis, erythema multiforme, pityriasis rosea, and urticaria [7, 8].

Given the widespread global vaccine rollout, maintaining pharmacovigilance is essential to ensure the continued safety and effectiveness of COVID-19 vaccines. Understanding the mechanisms underlying such complications and identifying them promptly will help safeguard patient health. These observations not only enhance our knowledge of vaccine-related reactions, but also provide meaningful insights for the development and monitoring of future vaccination programs.

## Methodology

This case report was prepared in accordance with the CAse REports (CARE) guidelines [9]. A systematic review was subsequently performed following the Preferred Reporting Items for Systematic Reviews and Meta-Analyses (PRISMA) framework.

A comprehensive literature search was conducted in PubMed, Scopus, Embase, and Web of Science in March 2024 to identify cases of bullous pemphigoid associated with COVID-19 vaccination. The search strategy included the following terms:

- **PubMed:** ((“covid 19 vaccines”[MeSH Terms] OR (“covid 19”[All Fields] AND “vaccines”[All Fields]) OR “covid 19 vaccines”[All Fields] OR “covid 19 vaccine”[All Fields]) AND (“pemphigoid, bullous”[MeSH Terms] OR (“pemphigoid”[All Fields] AND “bullous”[All Fields]) OR “bullous pemphigoid”[All Fields] OR ("bullous"[All Fields] AND “pemphigoid”[All Fields])))
- **EMBASE:** (‘covid-19 vaccine’/exp OR ‘covid-19 vaccine’ OR ((‘covid 19’/exp OR ‘covid 19’) AND (‘vaccine’/exp OR vaccine))) AND ‘bullous pemphigoid’:ti
- **Scopus:** (TITLE-ABS-KEY (covid-19 AND vaccine) AND TITLE (bullous AND pemphigoid))
- **Web of Science:** COVID-19 vaccine (All Fields) AND bullous pemphigoid (Title)

A total of 111 records were identified, with 57 duplicates removed. The remaining 54 articles underwent screening.

**Inclusion criteria:**

1. Case reports, case series, or letters that documented bullous pemphigoid following COVID-19 vaccination.
2. Biopsy-confirmed diagnosis.

**Exclusion criteria:**

1. Patients with a known history of bullous pemphigoid prior to vaccination.
2. Cases reported to be in remission.

**Fig. 1:**
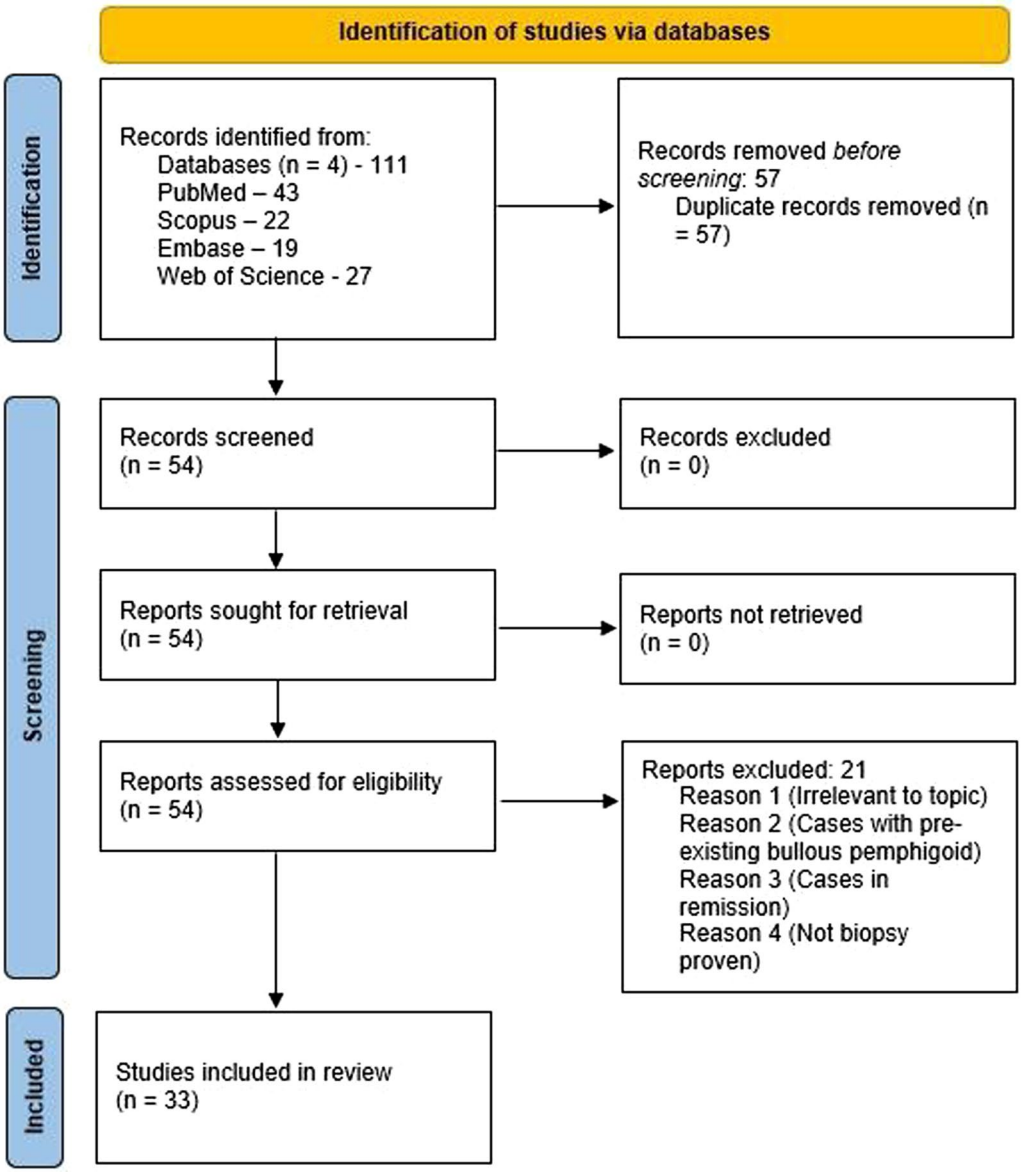
Flow diagram detailing study selection process (original figure by the authors)

Critical appraisal of included studies was performed using the Joanna Briggs Institute (JBI) critical appraisal checklist, resulting in the selection of 33 studies for data extraction. Figure 1 shows the PRISMA flow diagram for the study selection process.

## Case presentation

**Fig. 2:**
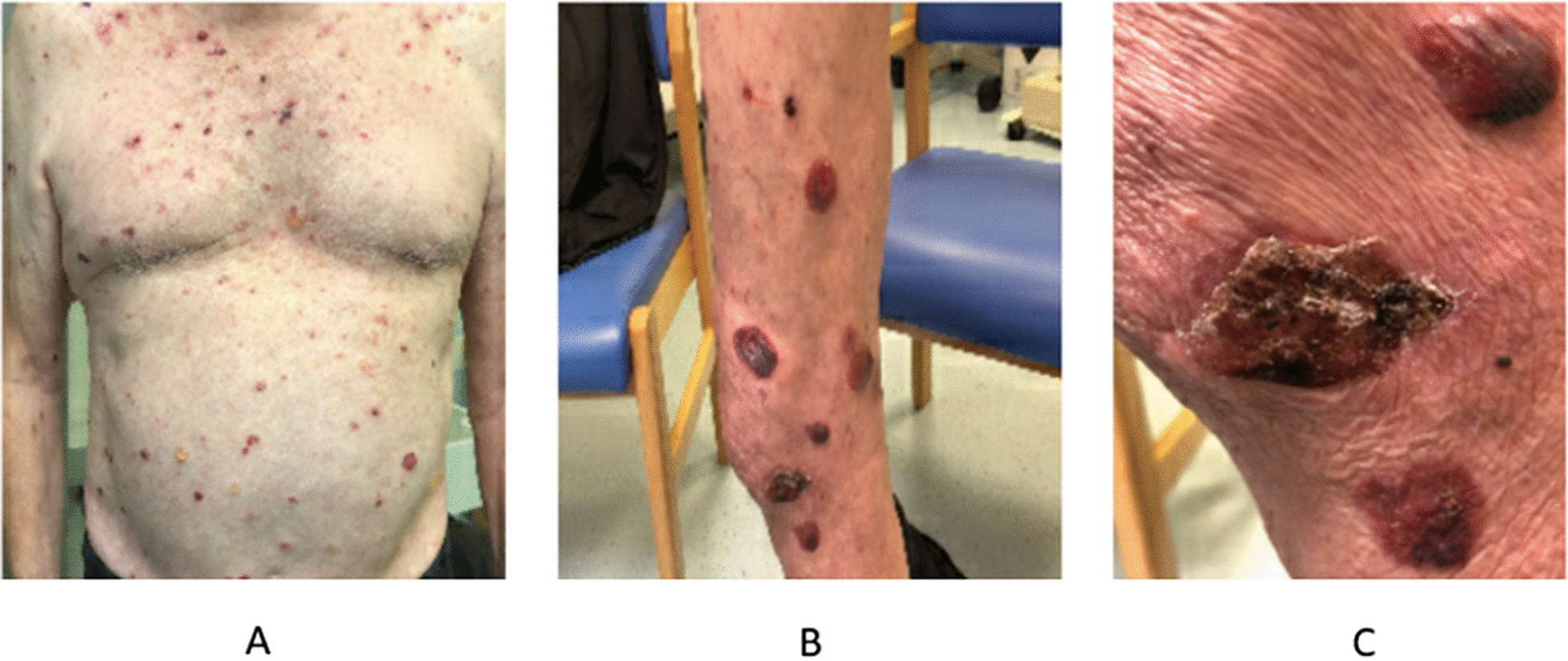
Initial presentation to the dermatology clinic; (**A**) erythematous papules and plaques involving chest and trunk, (**B**) erythematous plaques and crusted erosions involving lower limbs, (**C**) lesions around knee

**Fig. 3:**
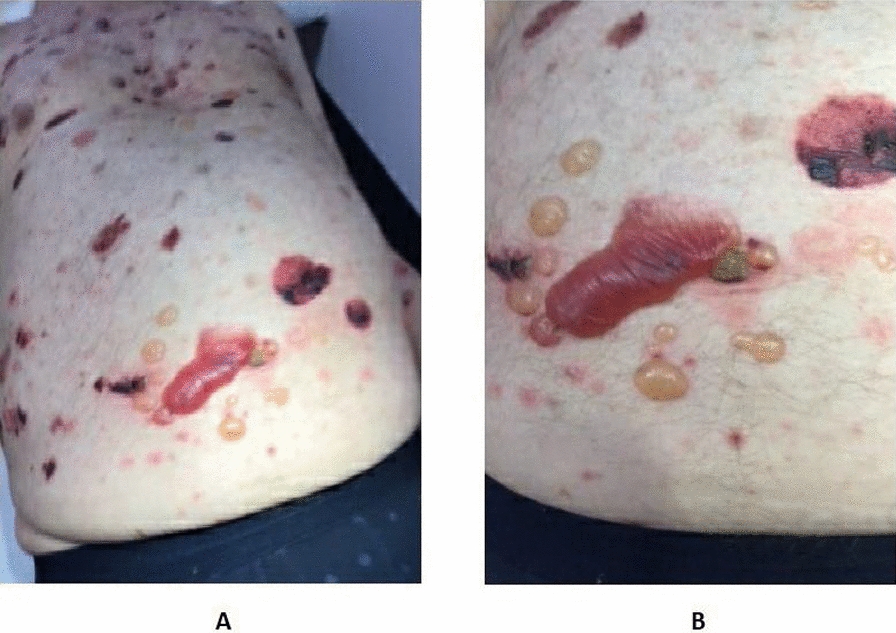
Worsening of lesions over time; (**A**) bullae in chest and abdomen, (**B**) bullae in mid-lower trunk

An elderly British man in his 80s, with history of type 2 diabetes mellitus, was referred by the general practitioner to the dermatology clinic for new onset blistering lesions on both his lower limbs, 21 days following the third dose of COVID vaccine (Moderna mRNA vaccine). His first two doses were AstraZeneca viral vector vaccines, following which he was fine. He had no past medical history related to any dermatological conditions apart from eczema when he was born and psoriasis during his teenage years, both of which subsided soon after diagnosis and using topical ointments, and never re-occurred. Neither he nor his family members have any past history of autoimmune diseases. He has no past medical history other than type 2 diabetes mellitus. Patient attributed his lesions to the vaccine he had received because he felt the timing and nature of lesions was too odd to be coincidental. The initial symptom was itching in lower limbs which then progressed to the development of erythematous patches in the lower limbs. Over a period of a few days, he was noted to have widespread erythematous papules and plaques on his trunks and both lower limbs on physical examination. He also had crusted erosions on the lower limbs (Fig. 2A–C) and was started on dermovate ointment (topical corticosteroid), emollients and doxycycline (antibiotic) 100 mg daily in the dermatology clinic to be continued for a period of 4 months. As time progressed, the lesions worsened, with development of multiple bullae over chest and abdomen (Fig. 3A, B) and he was also initiated on a weaning regimen of oral prednisolone (steroids) starting from 40 mg daily.

**Fig. 4:**
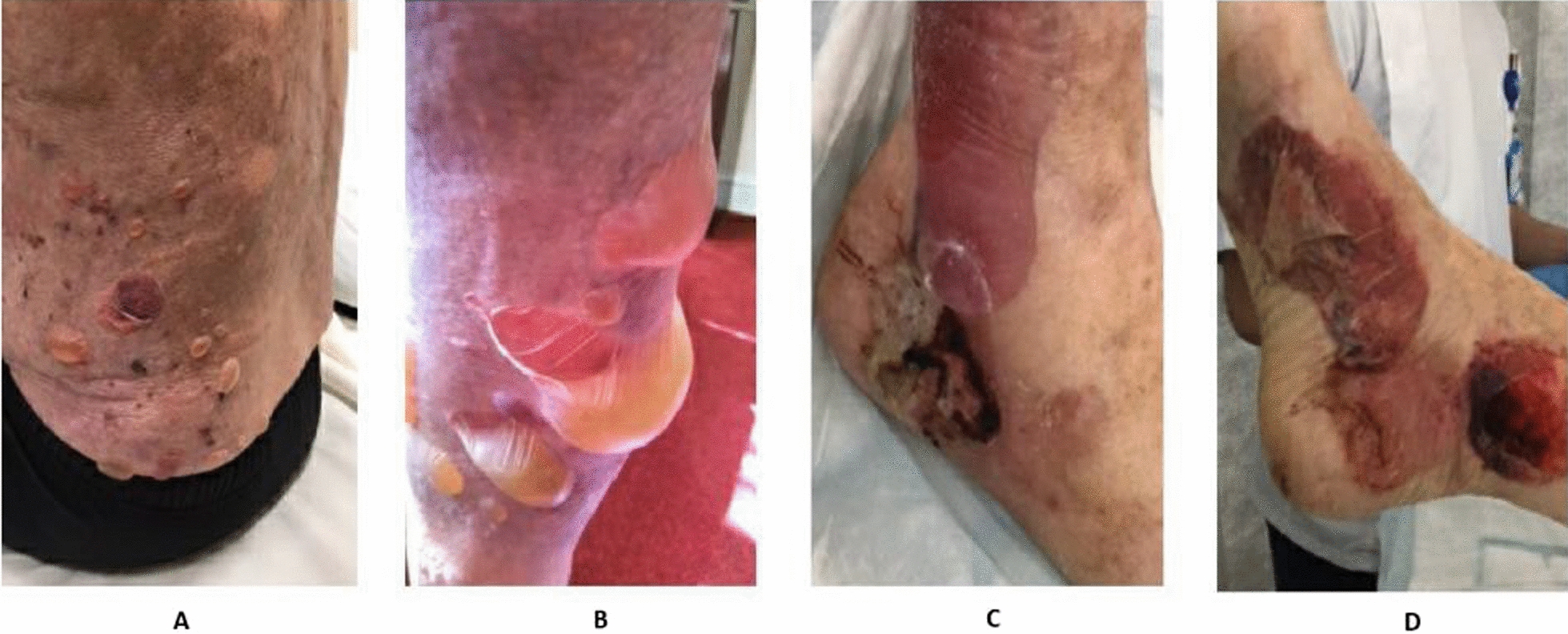
Flareup warranting hospital admission; (**A**) bullous lesions on left lower back, (**B**) bullous lesions on lower limbs, (**C**, **D**) bullae on foot

Pemphigoid antibody testing turned to be positive. Two 4 mm punch biopsy specimens were taken from chest wall and the histology from the biopsy confirmed bullous pemphigoid. Direct immunofluorescence showed features of a linear deposition of immunoglobulin (Ig)G and C3 at the basement membrane zone, consistent with the diagnosis. Despite him being on the weaning dose steroid regimen, there was a sudden severe flareup at 12 months. On physical examination, there was extensive body surface area involvement and large tense blisters on trunks and all limbs (Fig. 4A–D). The skin was extremely sore and there was visible peeling of skin, however, mucosal membranes were spared. Owing to the severity of the flareup, he was admitted to the hospital and had his wounds managed as an inpatient. Some of his large bullae were deflated with a sterile needle. The patient was responding to the treatment and wound care was provided in the hospital. Blood tests and further workup was done by the dermatology team to start him on mycophenolate mofetil (immunosuppressant therapy) 500 mg twice a day for a duration of 3 months for further management of his severe bullous pemphigoid.

### Outcome and follow-up

**Fig. 5:**
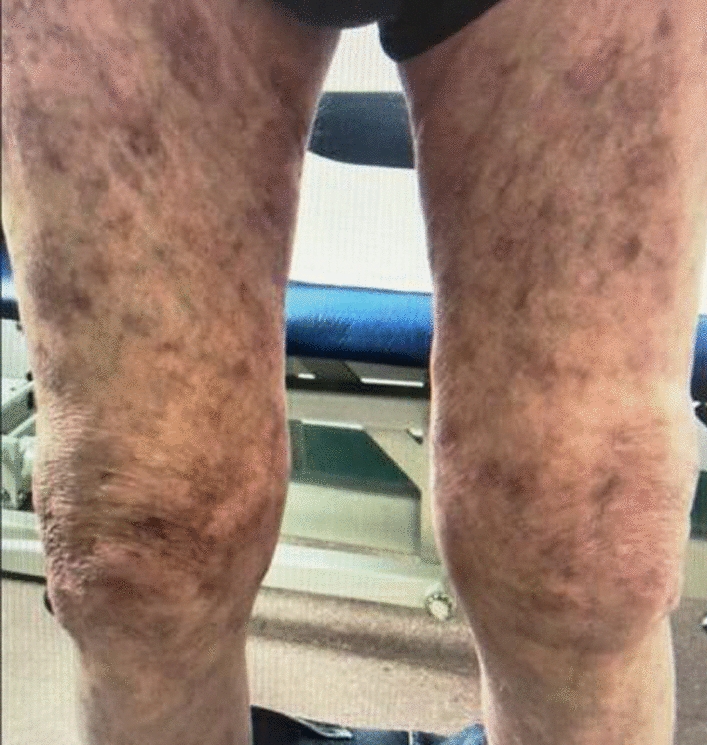
Resolution of lesions in lower limbs

The outcome of the treatment and wound care was highly satisfactory as the patient clinically improved (Fig. 5). The lesions stopped spreading and healing process was heading in the right direction. He will be followed up regularly in the dermatology clinic where he will be commenced on immunosuppressants.

### Strengths and limitations of the case report

The strengths of this case are:

- It establishes a certain degree of correlation between the vaccine and the condition using some of the scientific Bradford Hill criteria, such as temporality, consistency, and plausibility.
- Includes previously reported articles suggesting similar links in the discussion.
- Using the Naranjo Adverse Drug Reaction Probability Scale, a probability score of 4 was achieved, which implies a possible adverse drug reaction.

The limitation of this case report is that the patient is at an age where the condition can manifest, making it difficult to say for sure that this could have been triggered by the vaccine. However, given the multiple cases reported, the possibility does need to be considered for future vigilance and research regarding the extent of correlation between COVID vaccine and autoimmune disorders.

## Discussion/results

Our literature review yielded 33 articles consisting of 50 patients, which reported cases of bullous pemphigoid secondary to COVID-19 vaccination (Table 1).

**Table 1:** Literature review of 33 articles that reported COVID vaccine induced bullous pemphigoid

Sl no.	Title	Age	Sex	Type of vaccine	Dose	Duration between vaccination and appearance of symptoms	Blister location	Ab BP 180	Ab BP 230	Management	Dura treatment
1	Bullous drug eruption after second dose of mRNA-1273 (Moderna) COVID-19 vaccine: case report [10]	66	M	Moderna (mRNA-1273)	Second	< 24 hours	Generalized	Negative	Negative	Oral steroids + analgesics	7–14 days
2	Bullous pemphigoid associated with prodromal-phase by repeated COVID-19 vaccinations [11]	72	M	Pfizer-BioNTech (BNT162b2)	Third	24 hours–7 days	Generalized	Positive	NA	Oral steroids	Not specified
3	New-Onset pemphigoid gestationis following COVID-19 vaccination [12]	36	F	Pfizer-BioNTech (BNT162b2)	Second	> 7 days	Generalized	Positive	Negative	Oral steroids	Not specified
4	Autoimmune skin disorders and SARS-CoV-2 vaccination—A meta-analysis [13]	66	M	Pfizer-BioNTech (BNT162b2)	First	> 7 days	Generalized	Positive	NA	Topical steroids	Not specified
5	Mucous membrane pemphigoid following the administration of COVID-19 vaccine [14]	74	F	Pfizer-BioNTech (BNT162b2)	First	> 7 days	Oral	NA	NA	Oral steroids + antibiotics	> 14 days
6	SARS-CoV-2 vaccine-triggered conversion from systemic lupus erythematosus (SLE) to bullous SLE and dipeptidyl peptidase 4 inhibitors-associated bullous pemphigoid [15]	71	M	Pfizer-BioNTech (BNT162b2)	Second	> 7 days	Generalized	Positive	NA	Steroid injection/systematic therapy	Not specified
7	Oral mucous membrane pemphigoid after SARS-CoV-2 vaccination [16]	72	F	Pfizer-BioNTech (BNT162b2)	Third	> 7 days	Oral	Positive	Negative	Oral steroids	> 14 days
8	Bullous pemphigoid after vaccination with the inactivated severe acute respiratory syndrome coronavirus 2 vaccine: two cases in China [17]	67	F	Inactivated vaccines (CoronaVac, Sinopharm, and others)	First	24 hours–7 days	Generalized	Positive	NA	Oral steroids + antibiotics	7–14 days
		66	F	Inactivated vaccines (CoronaVac, Sinopharm, and others)	First	> 7 days	Generalized	Positive	NA	Oral steroids + antibiotics	7–14 days
9	A new eruption of bullous pemphigoid following mRNA COVID-19 vaccination [18]	70	M	Pfizer-BioNTech (BNT162b2)	Second	24 hours–7 days	Generalized	Positive	Negative	Steroid injection/systematic therapy	> 14 days
10	Bullous pemphigoid after inactivated COVID-19 vaccination: case report [19]	23	M	Inactivated vaccines (CoronaVac, Sinopharm, and others)	Third	24 hours–7 days	Generalized	Positive	Positive	Steroid injection/systematic therapy	7–14 days
		81	M	Inactivated vaccines (CoronaVac, Sinopharm, and others)	Third	> 7 days	Generalized	Positive	NA	Steroid injection/systematic therapy	Not specified
11	A case of acquired hemophilia A and bullous pemphigoid following SARS-CoV-2 mRNA vaccination [20]	77	M	Moderna (mRNA-1273)	Second	> 7 days	Trunk	NA	NA	Steroid injection/systematic therapy	> 14 days
12	Absolving COVID-19 vaccination of autoimmune bullous disease onset [21]	75	M	Pfizer-BioNTech (BNT162b2)	First	24 hours–7 days	Generalized	NA	NA	Topical steroids	Not specified
13	Incident bullous pemphigoid in a psoriatic patient following mRNA-1273 SARS-CoV-2 vaccination [22]	39	M	Moderna (mRNA-1273)	First	> 7 days	Upper limbs	NA	NA	Oral steroids + antibiotics	7–14 days
14	Bullous pemphigoid in a young male after COVID-19 mRNA vaccine: a report and brief literature review [23]	46	M	Pfizer-BioNTech (BNT162b2)	First	> 7 days	Generalized	Positive	NA	Steroid injection/systematic therapy	> 14 days
15	Bullous pemphigoid after SARS-CoV-2 vaccination: spike-protein-directed immunofluorescence confocal microscopy and T-cell-receptor studies [24]	80	M	Pfizer-BioNTech (BNT162b2)	First	> 7 days	Lower limbs	Positive	Positive	Oral steroids	Not specified
		89	M	Pfizer-BioNTech (BNT162b2)	First	24 hours–7 days	Generalized	Positive	Positive	Oral steroids	Not specified
16	Bullous pemphigoid induced by the AstraZeneca COVID-19 vaccine [25]	77	M	AstraZeneca (ChAdOx1-S vaccine)	First	< 24 hours	Generalized	NA	NA	Topical steroids + antibiotics	Not specified
17	Biphasic bullous pemphigoid starting after first dose and boosted by second dose of mRNA-1273 vaccine in an 84-year-old female with polymorbidity and polypharmacy [26]	84	F	Moderna (mRNA-1273)	Second	> 7 days	Generalized	Positive	Positive	Not specified	Not specified
18	A case of bullous pemphigoid after the SARS-CoV-2 mRNA vaccine [27]	68	M	Pfizer-BioNTech (BNT162b2)	First	24 hours–7 days	Trunk	NA	NA	Topical steroids	Not specified
19	Case of bullous pemphigoid following coronavirus disease 2019 vaccination [28]	83	F	Pfizer-BioNTech (BNT162b2)	Second	24 hours–7 days	Generalized	Positive	NA	Steroid injection/systematic therapy	Not specified
20	Clinical and histopathological spectrum of delayed adverse cutaneous reactions following COVID-19 vaccination [29]	76	M	Pfizer-BioNTech (BNT162b2)	First	> 7 days	Lower limbs	NA	NA	Topical steroids + antibiotics	Not specified
		85	M	Moderna (mRNA-1273)	Second	> 7 days	Generalized	NA	NA	Steroid injection/systematic therapy	Not specified
21	Subepidermal blistering eruptions, including bullous pemphigoid, following COVID-19 vaccination [30]	97	F	Pfizer-BioNTech (BNT162b2)	Second	24 hours–7 days	Generalized	Positive	Positive	Topical steroids + antibiotics	> 14 days
		75	M	Pfizer-BioNTech (BNT162b2)	Second	> 7 days	Generalized	Positive	Negative	Oral steroids + antibiotics	> 14 days
		64	M	Pfizer-BioNTech (BNT162b2)	Second	> 7 days	Generalized	Positive	Positive	Topical steroids	> 14 days
		82	M	Pfizer-BioNTech (BNT162b2)	Second	< 24 hours	Generalized	Negative	Negative	Topical steroids	> 14 days
		95	F	Pfizer-BioNTech (BNT162b2)	First	24 hours–7 days	Generalized	Negative	Negative	Topical steroids + antibiotics	> 14 days
		87	M	Moderna (mRNA-1273)	Second	> 7 days	Generalized	Positive	Positive	Oral steroids + antibiotics	> 14 days
		42	F	Moderna (mRNA-1273)	Second	24 hours–7 days	Generalized	Positive	Positive	Steroid injection/systematic therapy	> 14 days
		85	M	Pfizer-BioNTech (BNT162b2)	First	24 hours–7 days	Generalized	Negative	Negative	Oral steroids	> 14 days
		83	F	Moderna (mRNA-1273)	First	> 7 days	Generalized	Negative	Negative	Steroid injection/systematic therapy	> 14 days
		66	F	Pfizer-BioNTech (BNT162b2)	First	24 hours–7 days	Generalized	Negative	Negative	Steroid injection/systematic therapy	> 14 days
		70	F	Moderna (mRNA-1273)	First	> 7 days	Generalized	Negative	Negative	Oral steroids	7–14 days
		83	F	Pfizer-BioNTech (BNT162b2)	Second	24 hours–7 days	Generalized	Negative	Negative	Oral steroids + antibiotics	> 14 days
		83	M	Pfizer-BioNTech (BNT162b2)	First	24 hours–7 days	Generalized	Negative	Negative	Steroid injection/systematic therapy	> 14 days
22	Bullous pemphigoid and COVID-19 vaccine [31]	78	F	Pfizer-BioNTech (BNT162b2)	Second	24 hours–7 days	Generalized	NA	NA	Steroid injection/systematic therapy	Not specified
23	COVID-19 vaccine-triggered bullous pemphigoid: two new cases from Saudi Arabia [32]	86	M	AstraZeneca (ChAdOx1-S vaccine)	First	> 7 days	Generalized	Positive	Positive	Topical steroids	> 14 days
		76	M	Pfizer-BioNTech (BNT162b2)	First	> 7 days	Generalized	Positive	Positive	Topical steroids	> 14 days
24	Bullous pemphigoid associated with Covid-19 vaccine in child: a case report [33]	11	M		First	24 hours–7 days	Generalized	NA	NA	Steroid injection/systematic therapy	> 14 days
25	COVID-19 vaccine induced bullous pemphigoid: case report and review of the literature [34]	41	F	Pfizer-BioNTech (BNT162b2)	First	> 7 days	Upper limbs	NA	NA	Oral steroids	7–14 days
26	New-onset bullous pemphigoid triggered by AstraZeneca COVID-19 vaccine [35]	49	F	AstraZeneca (ChAdOx1-S vaccine)	First	< 24 hours	Trunk	NA	NA	Oral steroids	Not specified
27	Bullous pemphigoid onset following Pfizer-BioNTech COVID-19 vaccine (COMIRNATY®): coincidence or a new emerging adverse effect? [36]	61	M	Pfizer-BioNTech (BNT162b2)	First	> 7 days	Oral	Positive	NA	Oral steroids	> 14 days
28	Bullous pemphigoid with subsequent milia en plaque following administration of the Pfizer-BioNTech COVID-19 vaccine [37]	68	F	Pfizer-BioNTech (BNT162b2)	First	24 hours–7 days	Upper limbs	Positive	Positive	Oral steroids + antibiotics	Not specified
29	Bullous pemphigoid after second dose of mRNA-(Pfizer- BioNTech) Covid-19 vaccine: a case report [38]	78	M	Pfizer-BioNTech (BNT162b2)	Second	24 hours–7 days	Extremities	NA	NA	Oral steroids + antibiotics	> 14 days
30	Bullous pemphigoid developed after the COVID-19 vaccine [39]	91	M	Pfizer-BioNTech (BNT162b2)	Second	24 hours–7 days	Generalized	Positive	Positive	Oral Steroids	Not specified
		75	M	Pfizer-BioNTech (BNT162b2)	Second	24 hours–7 days	Lower limbs	Negative	Positive	Oral steroids	Not specified
31	New-onset bullous pemphigoid after inactivated Covid-19 vaccine: synergistic effect of the Covid-19 vaccine and vildagliptin [40]	67	M	Inactivated vaccines (CoronaVac, Sinopharm, and others)	First	> 7 days	Generalized	NA	NA	Oral steroids	> 14 days
32	Bullous pemphigoid triggered by COVID-19 vaccine: rapid resolution with corticosteroid therapy [41]	83	M	Pfizer-BioNTech (BNT162b2)	First	> 7 days	Lower limbs	NA	NA	Oral steroids	> 14 days
33	Reply to “New-onset bullous pemphigoid after inactivated Covid-19 vaccine: synergistic effect of the Covid-19 vaccine and vildagliptin” [42]	85	M	Pfizer-BioNTech (BNT162b2)	Second	Not specified	Not specified	Positive	Positive	Oral steroids + antibiotics	Not specified

The mean age of the cohort was 70.85 years. Seven patients (14%) were of Asian origin, three (6%) were of Middle Eastern origin, and the rest were unspecified. The incidence of post-COVID-19-vaccine.induced bullous pemphigoid were higher among male individuals (32, 64%) compared with female individuals (18, 36%). In 27 (54%) of the cases, there was no previous history of any drug allergy, and in rest of the cases (23) it was not reported.

**Fig. 6:**
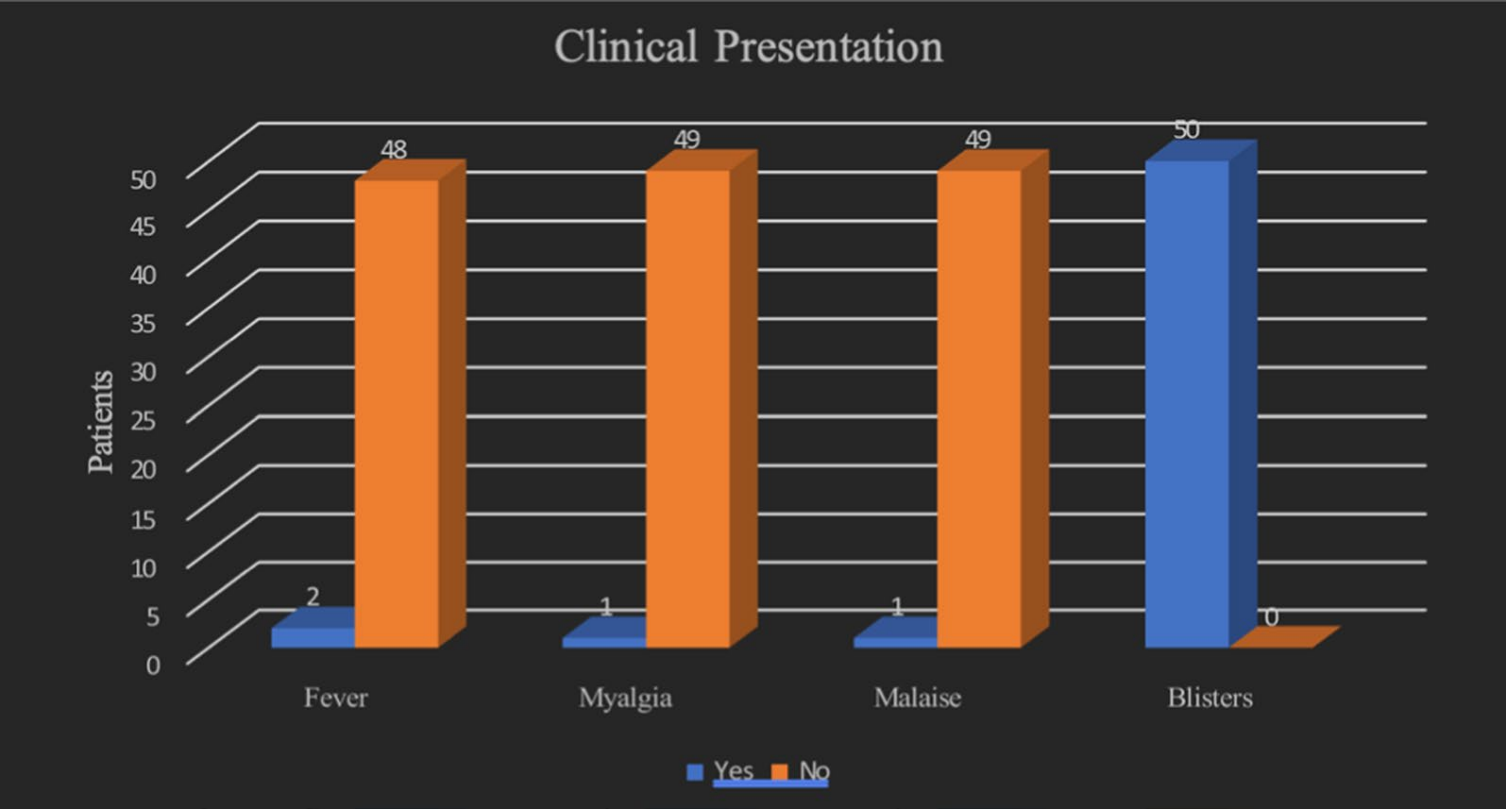
Clinical presentation of various cases of bullous pemphigoid triggered by COVID vaccine (original figure by the authors)

The Pfizer-BioNTech (BNT162b2) vaccine was seen to be associated with the majority of the cases: 32 (64%), followed by Moderna (mRNA-1273) in 9 cases (18%), inactivated vaccines (CoronaVac, Sinopharm, and others) in 5 cases (10%), and AstraZeneca (ChAdOx1-S vaccine) in 3 cases (6%). The majority of the cases were reported after receiving the first dose of the vaccine (26 cases, 52%), followed by the second dose, in which 20 (40%) cases were reported, and the least number after the third dose (4 cases, 8%). The duration between the appearance of symptoms and the administration of the vaccine was after the first week in 48% of cases, between 24 hours and 7 days in 42% of cases, and in less than 24 hours in 8% of cases. The common clinical features reported in the cohort were as following (Fig. 6): blisters in 100% of the patients, followed by other symptoms which were reported only in few patients, such as fever (two patients), myalgia (one patient), and malaise (one patient).

**Fig. 7:**
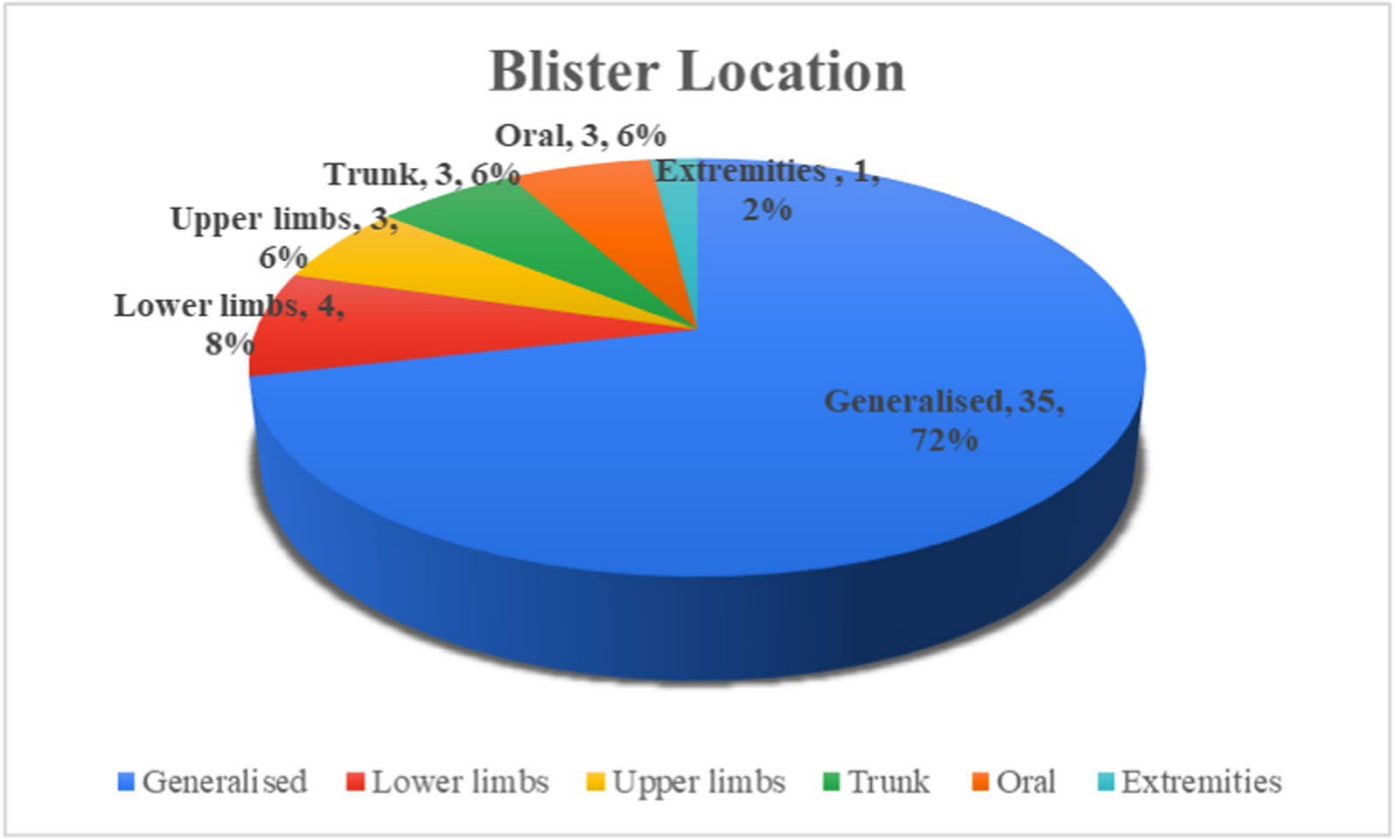
Blister location in the reported cases (original figure by the authors)

The location of the blisters in the reported cases were as follows (Fig. 7): generalized in 72% of the cases, lower limb in 8% of the cases, and 6% each in the lower limb, oral, and trunk. Mucous membrane was reported only in 8 (16%) cases, and in the majority of cases (40, 80%), there was no involvement of the mucous membrane.

**Fig. 8:**
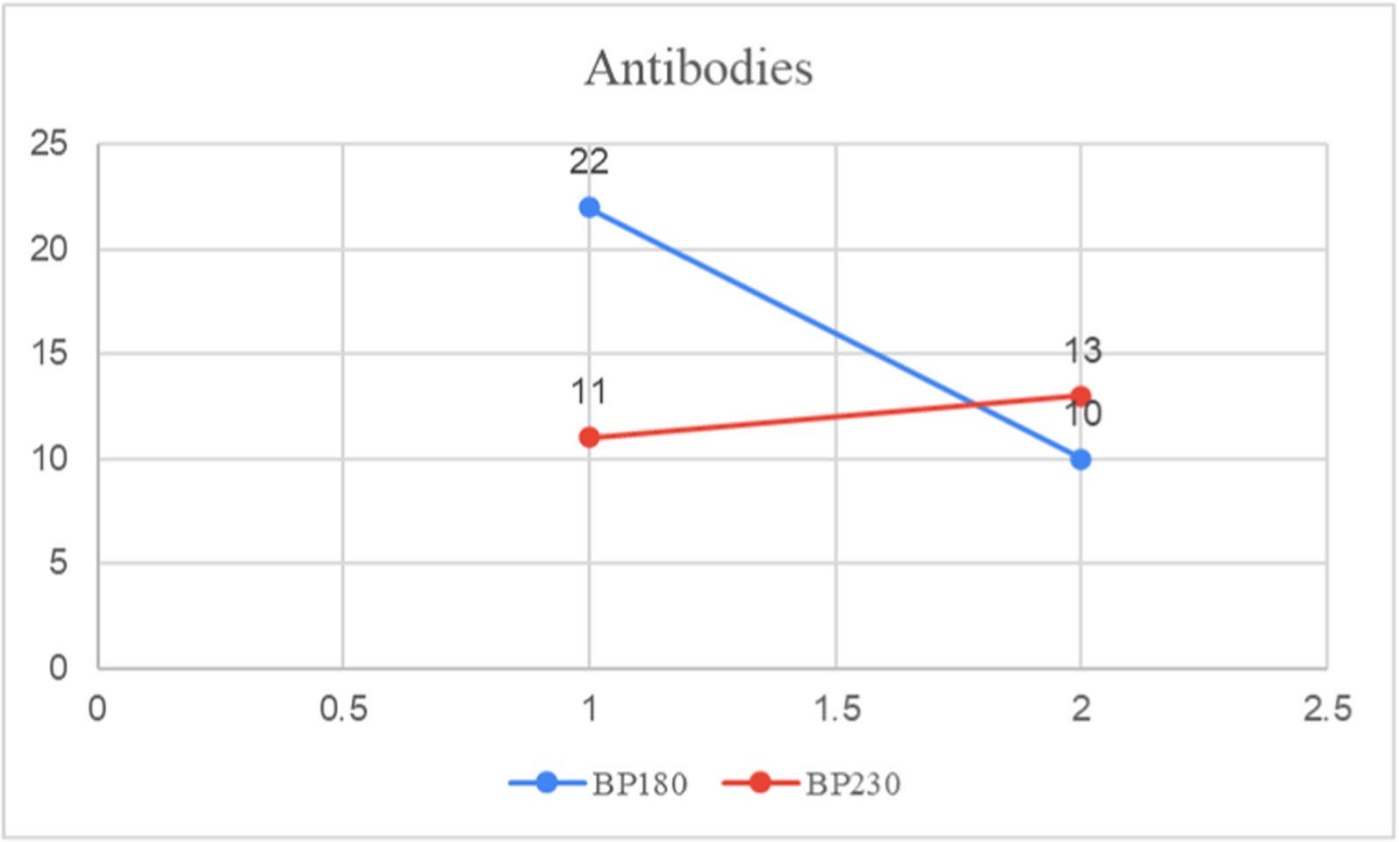
BP180 and BP230 antibody testing (original figure by the authors)

In the cases where antibody (Ab) testing was included in the investigation panel, BP180 testing was performed in 32 cases, yielding 22 positive results and 10 negative results. BP230 testing was conducted in 24 cases, with 11 positive results and 13 negative results (Fig. 8).

The majority of the cases were managed with systemic steroids (oral/intravenous) along with analgesics, and topical steroids along with analgesics (14 cases, 28%) each. Oral steroids along with antibiotics were used for management in 10 (20%) cases, topical steroids in 6 (12%), and topical steroids along with antibiotics in 4 (8%). In the majority of cases (48%), the mean duration of treatment was for more than 14 days, and in 7 cases (14%), it was between 7 and 14 days. Throughout the entire course of the pandemic, several cases of autoimmunity have been documented occurring in relation to COVID-19 vaccination. These included not only skin reactions, but also other autoimmune phenomena such as immune thrombocytopenia, myocarditis, Guillain-Barré syndrome, IgA nephropathy, and systemic lupus erythematosus [43]. The mechanism by which the vaccine causes autoimmune disorders has been explored, and one hypothesis is that the vaccine may induce autoimmunity in genetically predisposed individuals by stimulating a preexistent and subclinical autoreactivity against hemidesmosomal components [44]. Another proposed theory is that the vaccine-related autoimmune responses may include molecular mimicry (where vaccine components resemble human proteins and the immune system mistakenly attacks the human proteins), bystander activation (where immune cells become activated not directly by their specific antigen, but rather due to the presence of other activated immune cells or inflammatory signals), and epitope spreading [45]. However, despite the incidence of several skin reactions being reported, there are only speculations regarding the mechanism and it still remains unclear. The pathogenesis of COVID-vaccine-induced autoimmunity still warrants further research.

## Conclusion

Any new scientific development should be monitored for its own side effects. Bradford Hill criteria for causation includes nine principles that can be applied in such cases to establish a causal relationship between a presumed cause and an observed effect. Necessary precautions, monitoring for side effects, and prompt timely diagnosis can help prevent, or at the very least, treat complications at an earlier stage. While COVID-19 vaccines are essential in controlling the pandemic, awareness and understanding of their potential adverse effects, such as bullous pemphigoid, are critical in ensuring patient safety and improving therapeutic outcomes. There is scope for further research to establish the mechanism by which COVID vaccination causes occurrence of bullous pemphigoid.

## Data Availability

The data that support the findings of this article are available from the corresponding author upon reasonable request.

## References

[1] Velikova T, Georgiev T. SARS-CoV-2 vaccines and autoimmune diseases amidst the COVID-19 crisis. Rheumatol Int. 2021;41(3):509–18. 10.1007/s00296-021-04792-9. 33515320 10.1007/s00296-021-04792-9 PMC7846902

[2] Fiolet T, Kherabi Y, MacDonald CJ, *et al*. Comparing COVID-19 vaccines for their characteristics, efficacy and effectiveness against SARS-CoV-2 and variants of concern: a narrative review. Clin Microbiol Infect. 2022;28(2):202–21. 10.1016/j.cmi.2021.10.005. 34715347 10.1016/j.cmi.2021.10.005 PMC8548286

[3] Chen Y, Xu Z, Wang P, *et al*. New-onset autoimmune phenomena post-COVID-19 vaccination. Immunology. 2022;165(4):386–401. 10.1111/imm.13443. 34957554 10.1111/imm.13443

[4] Jara LJ, Vera-Lastra O, Mahroum N, *et al*. Autoimmune post-COVID vaccine syndromes: does the spectrum of autoimmune/inflammatory syndrome expand. Clin Rheumatol. 2022;41(5):1603–9. 10.1007/s10067-022-06149-4. 35378658 10.1007/s10067-022-06149-4 PMC8979721

[5] Rodríguez Y, Rojas M, Beltrán S, *et al*. Autoimmune and autoinflammatory conditions after COVID-19 vaccination. New case reports and updated literature review. J Autoimmun. 2022;132: 102898. 10.1016/j.jaut.2022.102898. 36041291 10.1016/j.jaut.2022.102898 PMC9399140

[6] Sanker V, Mylavarapu M, Gupta P, Syed N, Shah M, Dondapati VVK. Post COVID-19 vaccination medium vessel vasculitis: a systematic review of case reports. Infection. 2024. 10.1007/s15010-024-02217-w. 38483787 10.1007/s15010-024-02217-w

[7] Avallone G, Quaglino P, Cavallo F, *et al*. SARS-CoV-2 vaccine-related cutaneous manifestations: a systematic review. Int J Dermatol. 2022;61(10):1187–204. 10.1111/ijd.16063. 35141881 10.1111/ijd.16063 PMC9111829

[8] Shakoei S, Kalantari Y, Nasimi M, *et al*. Cutaneous manifestations following COVID-19 vaccination: a report of 25 cases. Dermatol Ther. 2022;35(8): e15651. 10.1111/dth.15651. 35716105 10.1111/dth.15651 PMC9349410

[9] Sohrabi C, Mathew G, Maria N, *et al*. The scare 2023 guideline: updating consensus surgical case report (SCARE) guidelines. Int J Surg. 2023;109(5): 1136. 10.1097/JS9.0000000000000373. 37013953 10.1097/JS9.0000000000000373 PMC10389401

[10] Kong J, Cuevas-Castillo F, Nassar M, *et al*. Bullous drug eruption after second dose of mRNA-1273 (Moderna) COVID-19 vaccine: case report. J Infect Public Health. 2021;14(10):1392–4. 10.1016/j.jiph.2021.06.021. 34294590 10.1016/j.jiph.2021.06.021 PMC8264280

[11] Yamamoto S, Koga H, Tsutsumi M, *et al*. Bullous pemphigoid associated with prodromal-phase by repeated COVID-19 vaccinations. J Dermatol. 2024;51(1):e6–7. 10.1111/1346-8138.16940. 37698074 10.1111/1346-8138.16940

[12] Mustin DE, Huffaker TB, Feldman RJ. New-onset pemphigoid gestationis following COVID-19 vaccination. Cutis. 2023;111(5):E2–4. 10.12788/cutis.0772. 37406312 10.12788/cutis.0772

[13] Hinterseher J, Hertl M, Didona D. Autoimmune skin disorders and SARS-CoV-2 vaccination—A meta-analysis. J Dtsch Dermatol Ges. 2023;21(8):853–61. 10.1111/ddg.15114. 37218538 10.1111/ddg.15114

[14] Rungraungrayabkul D, Rattanasiriphan N, Juengsomjit R. Mucous membrane pemphigoid following the administration of COVID-19 vaccine. Head Neck Pathol. 2023;17(2):587–8. 10.1007/s12105-023-01539-9. 36849670 10.1007/s12105-023-01539-9 PMC9970687

[15] Nakahara Y, Yamane M, Sunada M, *et al*. SARS-CoV-2 vaccine-triggered conversion from systemic lupus erythematosus (SLE) to bullous SLE and dipeptidyl peptidase 4 inhibitors-associated bullous pemphigoid. J Dermatol. 2023;50(2):162–5. 10.1111/1346-8138.16687. 36578130 10.1111/1346-8138.16687 PMC9880653

[16] Calabria E, Antonelli A, Lavecchia A, *et al*. Oral mucous membrane pemphigoid after SARS-CoV-2 vaccination. Oral Dis. 2024;30(2):782–3. 10.1111/odi.14468. 36516333 10.1111/odi.14468 PMC9878175

[17] Guo Z, Wang Y, Tang H, *et al*. Bullous pemphigoid after vaccination with the inactivated severe acute respiratory syndrome coronavirus 2 vaccine: two cases in China. Wound Manag Prev. 2022;68(11):22–5. 36493373

[18] Daines B, Madigan LM, Vitale PA, *et al*. A new eruption of bullous pemphigoid following mRNA COVID-19 vaccination. Dermatol Online J. 2022. 10.5070/D328458525. 36259862 10.5070/D328458525

[19] Zhang Y, Lang X, Guo S, *et al*. Bullous pemphigoid after inactivated COVID-19 vaccination: case report. Dermatol Ther. 2022;35(8): e15595. 10.1111/dth.15595. 35608483 10.1111/dth.15595 PMC9347905

[20] Fu PA, Chen CW, Hsu YT, *et al*. A case of acquired hemophilia A and bullous pemphigoid following SARS-CoV-2 mRNA vaccination. J Formos Med Assoc. 2022;121(9):1872–6. 10.1016/j.jfma.2022.02.017. 35321820 10.1016/j.jfma.2022.02.017 PMC8919791

[21] Russo R, Gasparini G, Cozzani E, *et al*. Absolving COVID-19 vaccination of autoimmune bullous disease onset. Front Immunol. 2022;13: 834316. 10.3389/fimmu.2022.834316. 35251024 10.3389/fimmu.2022.834316 PMC8895245

[22] Hung WK, Chi CC. Incident bullous pemphigoid in a psoriatic patient following mRNA-1273 SARS-CoV-2 vaccination. J Eur Acad Dermatol Venereol. 2022;36(6):e407–9. 10.1111/jdv.17955. 35073431 10.1111/jdv.17955

[23] Pauluzzi M, Stinco G, Errichetti E. Bullous pemphigoid in a young male after COVID-19 mRNA vaccine: a report and brief literature review. J Eur Acad Dermatol Venereol. 2022;36(4):e257–9. 10.1111/jdv.17891. 34928518 10.1111/jdv.17891

[24] Gambichler T, Hamdani N, Budde H, *et al*. Bullous pemphigoid after SARS-CoV-2 vaccination: spike-protein-directed immunofluorescence confocal microscopy and T-cell-receptor studies. Br J Dermatol. 2022;186(4):728–31. 10.1111/bjd.20890. 34773638 10.1111/bjd.20890 PMC8653321

[25] Agharbi FZ, Eljazouly M, Basri G, *et al*. Bullous pemphigoid induced by the AstraZeneca COVID-19 vaccine. Ann Dermatol Venereol. 2022;149(1):56–7. 10.1016/j.annder.2021.07.008. 34686374 10.1016/j.annder.2021.07.008 PMC8481089

[26] Schmidt V, Blum R, Möhrenschlager M. Biphasic bullous pemphigoid starting after first dose and boosted by second dose of mRNA-1273 vaccine in an 84-year-old female with polymorbidity and polypharmacy. J Eur Acad Dermatol Venereol. 2022;36(2):e88–90. 10.1111/jdv.17722. 34606112 10.1111/jdv.17722

[27] Young J, Mercieca L, Ceci M, *et al*. A case of bullous pemphigoid after the SARS-CoV-2 mRNA vaccine. J Eur Acad Dermatol Venereol. 2022;36(1):e13–6. 10.1111/jdv.17676. 34547137 10.1111/jdv.17676 PMC8661451

[28] Nakamura K, Kosano M, Sakai Y, *et al*. Case of bullous pemphigoid following coronavirus disease 2019 vaccination. J Dermatol. 2021;48(12):e606–7. 10.1111/1346-8138.16170. 34545973 10.1111/1346-8138.16170 PMC8652433

[29] Larson V, Seidenberg R, Caplan A, *et al*. Clinical and histopathological spectrum of delayed adverse cutaneous reactions following COVID-19 vaccination. J Cutan Pathol. 2022;49(1):34–41. 10.1111/cup.14104. 34292611 10.1111/cup.14104 PMC8444807

[30] Tomayko MM, Damsky W, Fathy R, *et al*. Subepidermal blistering eruptions, including bullous pemphigoid, following COVID-19 vaccination. J Allergy Clin Immunol. 2021;148(3):750–1. 10.1016/j.jaci.2021.06.026. 34275656 10.1016/j.jaci.2021.06.026 PMC8280592

[31] Pérez-López I, Moyano-Bueno D, Ruiz-Villaverde R. Bullous pemphigoid and COVID-19 vaccine. Medicina Clínica (English Edition). 2021;157(10):e333–4. 34697598 10.1016/j.medcle.2021.05.004 PMC8529265

[32] Dawoud NM, Aslam H, Alshehri MA, Dawoud MM. COVID-19 vaccine-triggered bullous pemphigoid: two new cases from Saudi Arabia. Indian J Dermatol. 2023;68(5):590. 10.4103/ijd.ijd_519_23. 38099105 10.4103/ijd.ijd_519_23 PMC10718263

[33] Mulianto N, Hashfi AF. Bullous pemphigoid associated with Covid-19 vaccine in child: a case report. J Pak Assoc Dermatol. 2023;33(2):730–5.

[34] Üstün P, Satılmış A, Kılıç İİ, *et al*. COVID-19 Vaccine induced bullous pemphigoid: case report and review of the literature. J Turk Acad Dermatol. 2023;17(1):27–30.

[35] Chao YC, Liu KL. New-onset bullous pemphigoid triggered by AstraZeneca COVID-19 vaccine. Dermatol Sin. 2022;40(4):245–6. 10.4103/1027-8117.358000.

[36] Ailenei E, *et al*. Bullous pemphigoid onset following Pfizer-BioNTech COVID-19 vaccine (COMIRNATY): coincidence or a new emerging adverse effect? Br J Dermatol. 2022;186(6): e251. 10.1111/bjd.21608.

[37] 17th Medical Dermatology Meeting, 13 January 2022. Br J Dermatol. 2022;186(6).

[38] Alshammari F, Abuzied Y, Korairi A, *et al*. Bullous pemphigoid after second dose of mRNA-(Pfizer-BioNTech) covid-19 vaccine: a case report. Ann Med Surg. 2022;75: 103420. 10.1016/j.amsu.2022.103420. 10.1016/j.amsu.2022.103420 PMC8885466 35251600

[39] Linares-Navarro R, Ramírez GR, Perandones-González H, Rodríguez-Prieto MÁ. Bullous pemphigoid developed after the COVID-19 vaccine. Skinmed. 2023;21(3):200–2. 37634107

[40] Bostan E, Yel B, Akdogan N, Gokoz O. New-onset bullous pemphigoid after inactivated Covid-19 vaccine: synergistic effect of the Covid-19 vaccine and vildagliptin. Dermatol Ther. 2022;35(2): e15241. 10.1111/dth.15241. 34854184 10.1111/dth.15241

[41] Dell’Antonia M, Anedda S, Usai F, Atzori L, Ferreli C. Bullous pemphigoid triggered by COVID-19 vaccine: rapid resolution with corticosteroid therapy. Dermatol Ther. 2022;35(1): e15208. 10.1111/dth.15208. 34786801 10.1111/dth.15208 PMC8646458

[42] Maronese CA, Di Zenzo G, Genovese G, *et al*. Reply to “New-onset bullous pemphigoid after inactivated Covid-19 vaccine: synergistic effect of the Covid-19 vaccine and vildagliptin.” Dermatol Ther. 2022;35(6): e15496. 10.1111/dth.15496. 35388592 10.1111/dth.15496 PMC9111836

[43] Rodríguez Y, Rojas M, Beltrán S, *et al*. Autoimmune and autoinflammatory conditions after COVID-19 vaccination. New case reports and updated literature review. J Autoimmun. 2022. 10.1016/j.jaut.2022.102898. 36041291 10.1016/j.jaut.2022.102898 PMC9399140

[44] Pira A, Sinagra JLM, Moro F, *et al*. Autoimmune bullous diseases during COVID-19 pandemic: 2022 update on rituximab and vaccine. Front Med Lausanne. 2023. 10.3389/fmed.2023.1112823. 36744126 10.3389/fmed.2023.1112823 PMC9893122

[45] Huang X, Liang X, Zhang J, *et al*. Pemphigus during the COVID-19 epidemic: infection risk, vaccine responses and management strategies. J Clin Med. 2022;11(14): 3968. 10.3390/jcm11143968. 35887732 10.3390/jcm11143968 PMC9317200

